# Perinatal and sociodemographic factors at birth predicting conduct problems and violence to age 18 years: comparison of Brazilian and British birth cohorts

**DOI:** 10.1111/jcpp.12369

**Published:** 2014-12-04

**Authors:** Joseph Murray, Barbara Maughan, Ana M B Menezes, Matthew Hickman, John MacLeod, Alicia Matijasevich, Helen Gonçalves, Luciana Anselmi, Erika A G Gallo, Fernando C Barros

**Affiliations:** 1 Department of Psychiatry, University of CambridgeCambridge, UK; 2 MRC Social, Developmental and Genetic Psychiatry Centre, Institute of Psychiatry, Psychology & Neuroscience, King's College LondonLondon, UK; 3 Postgraduate Program in Epidemiology, Federal University of PelotasPelotas, Brazil; 4 School of Social and Community Medicine, University of BristolBristol, UK; 5 Department of Preventive Medicine, Faculty of Medicine, University of São PauloSão Paulo, Brazil; 6 Postgraduate Program in Health and Behavior, Universidade Católica de PelotasPelotas, Brazil

**Keywords:** Conduct problems, violence, risk factors, cohort study, middle-income country, ALSPAC

## Abstract

**Background:**

Many low- and middle-income countries have high levels of violence. Research in high-income countries shows that risk factors in the perinatal period are significant precursors of conduct problems which can develop into violence. It is not known whether the same early influences are important in lower income settings with higher rates of violence. This study compared perinatal and sociodemographic risk factors between Brazil and Britain, and their role in explaining higher rates of conduct problems and violence in Brazil.

**Methods:**

Prospective population-based birth cohort studies were conducted in Pelotas, Brazil (*N* = 3,618) and Avon, Britain (*N* = 4,103). Eleven perinatal and sociodemographic risk factors were measured in questionnaires completed by mothers during the perinatal period. Conduct problems were measured in questionnaires completed by mothers at age 11, and violence in self-report questionnaires completed by adolescents at age 18.

**Results:**

Conduct problems were predicted by similar risk factors in Brazil and Britain. Female violence was predicted by several of the same risk factors in both countries. However, male violence in Brazil was associated with only one risk factor, and several risk factor associations were weaker in Brazil than in Britain for both females and males. Almost 20% of the higher risk for conduct problems in Brazil compared to Britain was explained by differential exposure to risk factors. The percentage of the cross-national difference in violence explained by early risk factors was 15% for females and 8% for males.

**Conclusions:**

A nontrivial proportion of cross-national differences in antisocial behaviour are related to perinatal and sociodemographic conditions at the start of life. However, risk factor associations are weaker in Brazil than in Britain, and influences in other developmental periods are probably of particular importance for understanding male youth violence in Brazil.

## Introduction

Childhood behaviour problems and youth violence are major global health problems. In 2010, 5.8 million healthy life years were lost worldwide due to conduct disorder, and 25.5 million lost due to injuries resulting from interpersonal violence (Murray et al., 2012). Research on the development of persistent, serious antisocial behaviour has highlighted the importance of early life influences that affect neurological and psychosocial functioning, including health factors during pregnancy and birth, and deprived social environments in infancy (Liu, 2011; Moffitt, 1993; Murray, Irving, Farrington, Colman, & Bloxsom, 2010; Raine, 2002). Although major longitudinal studies have been conducted in high-income countries in Europe, North America, and Australasia, the highest levels of serious violence are found in low- and middle-income countries in Latin America and Africa (Murray, Cerqueira, & Kahn, 2013). New studies are needed to test whether violence has similar origins in these regions. The current study compares the associations of perinatal and sociodemographic characteristics at birth with conduct problems and violence between large birth cohorts in Brazil and Britain.

Brazil is a middle-income country with the fifth largest population worldwide. Health indicators, such as infant mortality and life expectancy have improved considerably in Brazil in recent decades, but major challenges remain (Victora et al., 2011). Across low- and middle-income countries, over 200 million children do not reach their developmental potential by age five because of intrauterine growth restriction, nutritional deficiencies, exposure to toxins, violence, and other health and social problems (Walker et al., 2007). These early life influences may contribute to elevated risk for behaviour problems in childhood and adolescence. Since the 1980s, violence has become a leading cause of death in Brazil. In 2010, 3% of the world's population lived in Brazil, but 13% of all homicides took place there – more homicides than in any other country.^1^ Recently, we found that maternal-reported childhood conduct problems and self-reported adolescent violence were significantly more prevalent in Brazil than in Britain (Murray et al., 2015). In the current study, we examine whether perinatal and sociodemographic factors at birth contribute to these high rates of behaviour problems in Brazil compared to Britain. If they do, it should be observed that: (a) children in Brazil are exposed to more risk factors; (b) risk factors predict conduct problems and violence in Brazil as well as in Britain; (c) statistically controlling for risk factors while comparing across countries at least partly explains the higher risk for conduct problems and violence in Brazil. We test whether these conditions are met in two large, well-matched, population samples in Brazil and Britain.

## Method

### Pelotas 1993 birth cohort, Brazil

The 1993 Pelotas Birth Cohort is an ongoing population-based study designed to investigate the effects of a wide range of influences on health and development. Pelotas is a city located in the extreme south of Brazil, with an estimated population of 345,179 inhabitants, 93% of whom live in the urban area. All births occurring in the five maternity clinics in Pelotas were monitored in 1993 (99% of births in Pelotas occurred in hospital). For the 5,265 children born alive, only 16 mothers could not be interviewed or refused to participate in the study. The 5,249 newborns whose mothers lived in the urban area were included in the cohort. Mothers were interviewed in a perinatal study and follow-up home visits were conducted in 2004–2005 (age 11) and clinic sessions in 2011–2012 (age 18). The detailed methodology of the study can be found elsewhere (Gonçalves et al., 2014; Victora et al., 2008). Each assessment was approved by the Research Ethics Committee of the Federal University of Pelotas School of Medicine. Participants provided written informed consent at each stage of the study.

### Avon longitudinal study of parents and children (ALSPAC), Britain

ALSPAC is a separate ongoing population-based study in Britain. ALSPAC recruited 14,541 pregnant women resident in Avon with expected dates of delivery 1st April 1991 to 31st December 1992; and, from age 7, continued to recruit children born in that area at that time until age 18. We used data on 14,762 live-born singleton or twin children; triplets and quads were excluded for reasons of confidentiality. The detailed methodology of ALSPAC can be found elsewhere (Boyd et al., 2013) and the study website contains details of all the data that are available through a fully searchable data dictionary (http://www.bris.ac.uk/alspac/researchers/data-access/data-dictionary/). Mothers completed questionnaires during pregnancy, after birth, and when children were age 11 years, and cohort members participated in clinic sessions at age 18. Ethical approval for the study was obtained from the ALSPAC Ethics and Law Committee and the Local Research Ethics Committees.

### Measures

#### Conduct problems at age 11

When children were age 11 years, parents (usually mothers) completed the Strengths and Difficulties Questionnaire (SDQ) for 4,423 children in Pelotas and 7,307 children in ALSPAC. The SDQ is a screening questionnaire assessing child mental health, including conduct problems (symptoms of conduct disorder and oppositional defiant disorder), in the previous 6 months; it was validated in Brazil by Fleitlich-Bilyk and Goodman (2004). The standard cut-off point used to identify ‘abnormal’ levels of conduct problems (>3), was applied in Pelotas and ALSPAC.

#### Violence at age 18

A confidential self-reported questionnaire asking about crimes committed in the previous 12 months was completed by 4,102 adolescents in ALSPAC clinic sessions at age 18. To use this instrument in clinics in Pelotas at age 18, questions were first translated into Brazilian Portuguese, then pilot tested among adolescent offenders (in a young offenders’ institution) and among adolescents in the community (in a public health clinic), adjusted by bilingual researchers, further pilot tested, and then back translated into English. Due to a printing error, the first 325 questionnaires (8% of 4,106 participants) in Pelotas were not usable. The current analyses of violence in Pelotas include the vast majority of participants (*N* = 3,618) with complete crime data, who are extremely similar to the subsample without crime data on all perinatal characteristics (see online supplement, Table S1). We used a summary variable for violence, coded positive if the participant reported at least one of four behaviours: stole from person with threat/force, assault, carried a weapon for fights or self-defence, used weapon. In Pelotas, official police, court and juvenile justice institution data were also collected. The association between self-reported violence and having an official record of violent crime committed at age 18 was strong (risk ratio = 5.2).

#### Perinatal risk factors

Risk factors were measured during perinatal assessments with mothers in Pelotas, and during pregnancy and perinatal assessments with mothers in ALSPAC. The following variables were measured in both studies, and dichotomised to maximise comparability: unplanned pregnancy (yes/no), mother ever smoked in pregnancy (yes/no), mother used alcohol in pregnancy (yes/no), maternal urinary infection during pregnancy (yes/no), intrauterine growth restriction (yes/no; referring to <10th percentile/≥10th percentile for gestational age and gender, according to the reference curve developed by Kramer et al. (2001)), and premature birth <37 weeks (yes/no). All perinatal variables (and sociodemographic variables) except planned pregnancy and alcohol use have been carefully compared between Pelotas and ALSPAC in previous work (Matijasevich et al., 2012). Planned pregnancy was measured in a similar single questionnaire item in both studies. Alcohol use referred to at least weekly drinking during pregnancy in Pelotas, and at least weekly drinking either at 18 weeks gestation or in the last 2 months of pregnancy in ALSPAC. In addition to individual risk factors, the cumulative number of perinatal risk factors was summed, up to six, for each child.

#### Sociodemographic risk factors

The following sociodemographic characteristics were measured in perinatal assessments with mothers in both studies: maternal age (<20 years/≥20 years), low maternal education (yes/no; referring in Pelotas to 0**–**8 vs. ≥9 years of schooling; referring in ALSPAC to qualified up to certificate of secondary qualification level, vs. qualified to at least vocational level, O-level, or A-level), marital status (single mother/with partner), three or more siblings (yes/no), family income (lowest quintile/second-fifth quintiles). For each child, the cumulative number of sociodemographic risk factors was summed, up to five.

#### Statistical analyses

The prevalence of risk factors was compared between Pelotas and ALSPAC using risk ratios. Risk ratios were also used to examine associations between perinatal/sociodemographic variables and conduct problems and violence within each study. Differences in risk ratios (interactions) between studies were tested using the method recommended by Altman and Bland (2003) and reported if *p *<* *.10.

To examine whether risk factors explained cross-national differences in conduct problems/violence, we used a merged data set and a dummy indicator for study (1 = Pelotas, 0 = ALSPAC). First, we calculated crude ratios comparing rates of behaviours between Pelotas and ALSPAC. For example, if the prevalence of conduct problems was 30% in Pelotas and 15% in ALSPAC, the Pelotas–ALSPAC ratio would be 2.0 = 30/15. Then, adjusted Pelotas–ALSPAC ratios were calculated adjusting for cumulative exposure to risk factors, using Poisson regression with robust standard errors (Barros & Hirakata, 2003). If exposure to risk factors explains cross-national differences in conduct problems/violence, adjusted ratios should be smaller than crude ratios.

Although Pelotas participants with valid crime data were almost identical to those missing crime data, in ALSPAC attrition because of nonlocation of participants or nonresponse was nonrandom (see Table S1). Therefore, we calculated multivariate models using multiple imputations for missing data (see supporting information for results using complete case analyses). In multiple imputations, fifty data sets (each including 2,645 Pelotas females, 2,603 Pelotas males, 7,176 ALSPAC females and 7,586, ALSPAC males) were created with the *mi impute chained* command in STATA 12.1 (the programme used for all analyses). The following variables were used in the imputation process: study, participant sex, all perinatal variables, conduct problems, violence and age at measurement of outcomes (months). The cumulative numbers of perinatal and sociodemographic risk factors were calculated in each data set after imputation.

## Results

### Prevalence of perinatal and sociodemographic risk factors at birth

Newborns in Pelotas were more likely than in ALSPAC to have been conceived in an unplanned pregnancy, had mothers who smoked in pregnancy, had mothers who had a urinary infection in pregnancy, been born prematurely, had a young and single mother, and had three or more siblings (Table1).^2^ Children in Pelotas were also exposed to a higher cumulative number of perinatal and sociodemographic risk factors than in ALSPAC.

**Table 1:** Prevalence of perinatal and sociodemographic characteristics in Pelotas and ALSPAC

	Pelotas females	ALSPAC females	Study comparison	Pelotas males	ALSPAC males	Study comparison
	N (%)	N (%)	RR (95% CI)	N (%)	N (%)	RR (95% CI)
Unplanned pregnancy						
Yes	1,488 (56.3)	1,938 (30.5)	1.8 (1.8–1.9)	1,464 (56.3)	2,105 (31.0)	1.8 (1.7–1.9)
No	1,156 (43.7)	4,410 (69.5)	*p < *.001	1,138 (43.7)	4,676 (69.0)	*p < *.001
Ever smoked in pregnancy						
Yes	909 (34.4)	1,221 (23.0)	1.5 (1.4–1.6)	843 (32.4)	1,418 (25.3)	1.3 (1.2–1.4)
No	1,736 (65.6)	4,081 (77.0)	*p < *.001	1,760 (67.6)	4,195 (74.7)	*p < *.001
Alcohol use in pregnancy						
Yes	144 (5.4)	430 (7.8)	0.7 (0.6–0.8)	123 (4.7)	475 (8.1)	0.6 (0.5–0.7)
No	2,501 (95.6)	5,081 (92.2)	*p *<* *.001	2,480 (95.3)	5,388 (91.9)	*p *<* *.001
Urinary infection in pregnancy						
Yes	890 (34.2)	680 (13.6)	2.5 (2.3–2.7)	836 (33.0)	749 (14.1)	2.3 (2.2–2.6)
No	1,715 (65.8)	4,331 (86.4)	*p < *.001	1,698 (67.0)	4,573 (85.9)	*p < *.001
Intrauterine growth restriction						
Yes	235 (9.1)	567 (8.4)	1.1 (0.9–1.3)	252 (9.8)	662 (9.1)	1.1 (0.9–1.2)
No	2,362 (91.0)	6,216 (91.6)	*p = *.285	2,315 (90.2)	6,604 (90.9)	*p = *.290
Preterm birth (<37 weeks)						
Yes	304 (11.7)	373 (5.5)	2.1 (1.8–2.5)	285 (11.1)	512 (7.1)	1.6 (1.4–1.8)
No	2,297 (88.3)	6,410 (94.5)	*p < *.001	2,285 (88.9)	6,754 (93.0)	*p < *.001
**Number of perinatal risk factors**	**M = 1.5** ***SD* = 1.1**	**M = 0.8** ***SD* = 0.9**		**M = 1.5** ***SD* = 1.0**	**M = 0.9** ***SD* = 0.9**	
Maternal age						
<20	440 (16.6)	328 (4.8)	3.4 (3.0–3.9)	475 (18.3)	327 (4.5)	4.1 (3.5–4.6)
≥20	2,205 (83.4)	6,455 (95.2)	*p < *.001	2,127 (81.7)	6,939 (95.5)	*p < *.001
Maternal education						
Low	764 (28.9)	1,196 (19.9)	1.5 (1.3–1.6)	707 (27.2)	1,316 (20.5)	1.3 (1.2–1.4)
Medium-high	1,879 (71.1)	4,827 (80.1)	*p < *.001	1,895 (72.8)	5,118 (79.6)	*p < *.001
Marital status						
Single mother	307 (11.6)	123 (2.1)	5.5 (4.4–6.7)	342 (13.1)	167 (2.7)	4.8 (4.0–5.8)
With partner	2,338 (88.4)	5,654 (97.9)	*p < *.001	2,261 (86.9)	5,970 (97.3)	*p < *.001
Three or more siblings						
Yes	349 (13.2)	364 (5.9)	2.2 (2.0–2.6)	381 (14.6)	392 (5.9)	2.5 (2.2–2.8)
No	2,296 (86.8)	5,824 (94.1)	*p < *.001	2,222 (85.4)	6,233 (94.1)	*p < *.001
Family income						
Lowest quintile	516 (19.9)	934 (19.7)	1.0 (0.9–1.1)	514 (20.2)	1,017 (20.3)	1.0 (0.9–1.1)
Second-fifth quintile	2,073 (80.1)	3,802 (80.3)	*p = *.830	2,033 (79.8)	4,000 (79.7)	*p = *.926
**Number of sociodemographic risks**	**M = 0.9** ***SD* = 1.0**	**M = 0.4** ***SD* = 0.7**		**M = 0.9** ***SD* = 0.9**	**M = 0.4** ***SD* = 0.7**	

Note: % = column per cent; RR = risk ratio comparing proportion with risk factor in Pelotas and ALSPAC; CI = confidence interval; M = mean; *SD* = standard deviation. *p *<* *.001 for all *t*-tests comparing mean perinatal/sociodemographic risks between Pelotas and ALSPAC, for females and males.

### Associations between perinatal and sociodemographic risk factors and conduct problems and violence

Rates of conduct problems among males were 33.9% in Pelotas compared with 8.5% in ALSPAC, and rates of violence were 22.6% in Pelotas and 11.0% in ALSPAC (Murray et al., 2015). Among females, rates of conduct problems were 28.7% in Pelotas and 6.0% in ALSPAC, and rates of violence were 8.9% in Pelotas and 3.0% in ALSPAC. Tables2 and 3 show that the prevalence of conduct problems and violence was also higher in Pelotas than in ALSPAC across all categories of risk factors, for both sexes.

**Table 2:** Perinatal and sociodemographic characteristics and risk for conduct problems at age 11

	Pelotas females conduct problems		ALSPAC females conduct problems		Pelotas males conduct problems		ALSPAC males conduct problems	
	%	RR (95% CI)	%	RR (95% CI)	%	RR (95% CI)	%	RR (95% CI)
Unplanned pregnancy								
Yes	32.4	1.3 (1.2–1.5)	6.6	1.1 (0.8–1.5)	37.1	1.2 (1.1–1.4)	10.9	1.5 (1.2–1.9)
No	24.0	*p < .001*	5.9	*p = *.427	29.9	*p < *.001	7.4	*p = *.001
Ever smoked in pregnancy								
Yes	39.2	1.7 (1.5–1.9)	10.0	2.1 (1.5–2.8)	40.0	1.3 (1.1–1.5)	13.2	1.9 (1.5–2.5)
No	23.2	*p < *.001	4.8	*p < *.001	30.9	*p < *.001	6.8	*p *<* *.001
Alcohol use in pregnancy								
Yes	36.2	1.3 (1.0–1.6)	5.1	0.8 (0.5–1.5)	48.5	1.5 (1.2–1.8)	8.7	1.1 (0.7–1.6)
No	28.2	*p *=* *.053	5.7	*p *=* *.633	33.2	*p *=* *.002	7.9	*p *=* *.646
Urinary infection in pregnancy								
Yes	33.6	1.3 (1.1–1.5)	7.5	1.4 (0.9–2.0)	35.9	1.1 (1.0–1.2)	8.9	1.1 (0.8–1.6)
No	25.9	*p < *.001	5.5	*p = *.124	32.5	*p = *.126	8.1	*p = *.598
Intrauterine growth restriction								
Yes	34.3	1.2 (1.0–1.5)	6.1	1.0 (0.6–1.6)	37.5	1.1 (0.9–1.3)	11.7	1.5 (1.0–2.1)
No	28.0	*p = *.057	6.1	*p = *.988	33.7	*p = *.267	8.1	*p = *.037
Preterm birth (<37 weeks)								
Yes	28.9	1.0 (0.8–1.2)	5.8	0.9 (0.5–1.8)	40.0	1.2 (1.0–1.4)	9.3	1.1 (0.7–1.8)
No	28.6	*p = *.908	6.2	*p = *.848	33.5	*p = *.073	8.3	*p = *.587
**Number of perinatal risk factors**		**1.3 (1.2–1.3)** ***p < *.001**		**1.3 (1.1–1.5)** ***p < *.001**		**1.2 (1.1–1.2)** ***p < *.001**		**1.3 (1.2–1.5)** ***p < *.001**
Maternal age								
<20	39.4	1.5 (1.3–1.7)	11.1	1.8 (0.9–3.6)	40.9	1.3 (1.1–1.4)	17.7	2.2 (1.3–3.8)
≥20	26.5	*p < .001*	6.0	*p = *.075	32.4	*p = *.001	8.2	*p = *.007
Maternal education								
Low	36.5	1.4 (1.3–1.6)	8.4	1.5 (1.1–2.2)	43.9	1.5 (1.3–1.6)	12.9	1.7 (1.3–2.3)
Medium-high	25.5	*p < .001*	5.5	*p *=* *.016	30.2	*p < *.001	7.5	*p < *.001
Marital status								
Single mother	37.8	1.4 (1.2–1.6)	13.6	2.4 (1.1–5.0)	40.7	1.2 (1.1–1.4)	17.2	2.2 (1.2–3.9)
With partner	27.6	*p = *.001	5.8	*p = *.028	32.9	*p = *.010	7.8	*p = *.009
Three or more siblings								
Yes	33.7	1.2 (1.0–1.4)	10.1	1.7 (1.0–2.9)	39.7	1.2 (1–1.4)	9.2	1.1 (0.7–1.9)
No	27.9	*p = *.043	5.9	*p = *.039	32.9	*p = *.019	8.3	*p = *.673
Family income								
Lowest quintile	38.6	1.5 (1.3–1.7)	9.3	1.7 (1.3–2.4)	42.3	1.3 (1.2–1.5)	11.9	1.6 (1.2–2.1)
Second-fifth quintile	26.0	*p < .001*	5.3	*p = *.001	31.4	*p < *.001	7.3	*p < *.001
**Number of sociodemographic risks**		**1.3 (1.2–1.4)** ***p < *.001**		**1.4 (1.2–1.7)** ***p < *.001**		**1.2 (1.2–1.3)** ***p < *.001**		**1.5 (1.3–1.7)** ***p < *.001**

Note: % = row per cent of children with conduct problems; RR = risk ratio; CI = confidence interval.

**Table 3:** Perinatal and sociodemographic characteristics and risk for violence at age 18

	Pelotas females violence		ALSPAC females violence		Pelotas males violence		ALSPAC males violence	
	%	RR (95% CI)	%	RR (95% CI)	%	RR (95% CI)	%	RR (95% CI)
Unplanned pregnancy								
Yes	10.6	1.5 (1.1–2.1)	4.5	2.0 (1.2–3.4)	24.1	1.2 (1.0–1.4)	12.6	1.2 (0.9–1.7)
No	6.9	*p = *.006	2.3	*p = *.008	20.8	*p = *.100	10.3	*p = *.197
Ever smoked in pregnancy								
Yes	12.1	1.7 (1.2–2.2)	4.2	1.8 (1.0–3.4)	24.0	1.1 (0.9–1.3)	15.4	1.6 (1.2–2.3)
No	7.3	*p < *.001	2.3	*p = *.057	22.0	*p = *.359	9.4	*p = *.005
Alcohol use in pregnancy								
Yes	7.0	0.8 (1.4–1.6)	5.0	1.9 (0.9–4.0)	33.8	1.5 (1.1–2.1)	12.6	1.2 (0.9–1.7)
No	9.0	*p *=* *.486	2.6	*p *=* *.074	22.1	*p *=* *.015	10.3	*p *=* *.197
Urinary infection in pregnancy								
Yes	9.4	1.1 (0.8–1.5)	2.7	1.1 (0.5–2.5)	23.5	1.1 (0.9–1.3)	13.2	1.4 (0.9–2.1)
No	8.5	*p = *.554	2.5	*p = *.879	22.2	*p = *.538	9.7	*p = *.161
Intrauterine growth restriction								
Yes	11.1	1.3 (0.8–2.0)	3.1	1.1 (0.4–2.7)	20.5	0.9 (0.7–1.2)	11.5	1.0 (0.6–1.7)
No	8.8	*p = *.326	2.8	*p = *.837	23.0	*p = *.463	11.1	*p = *.888
Preterm birth (<37 weeks)								
Yes	8.7	1.0 (0.6–1.5)	*4.4*	1.6 (0.6–4.3)	21.4	1.0 (0.7–1.3)	*9.3*	0.8 (0.4–1.6)
No	9.0	*p = *.895	2.8	*p = *.357	22.9	*p = *.657	11.2	*p = *.559
**Number of perinatal risk factors**		**1.2 (1.1–1.4)** ***p *=* *.001**		**1.4 (1.0–1.9)** ***p *=* *.034**		**1.1 (1.0–1.2)** ***p = *.051**		**1.2 (1.0–1.4)** ***p *=* *.028**
Maternal age								
<20	10.2	1.2 (0.8–1.7)	0.0	N/A	25.2	1.1 (0.9–1.4)	8.0	0.7 (0.2–2.7)
≥20	8.7	*p = *.391	2.9	*p = *.302	22.1	*p = *.223	11.1	*p = *.620
Maternal education								
Low	11.2	1.4 (1.0–1.9)	5.8	2.4 (1.3–4.3)	23.6	1.1 (0.9–1.3)	10.5	1.0 (0.6–1.5)
Medium-high	8.1	*p = *.033	2.4	*p = *.003	22.3	*p = *.575	11.0	*p = *.832
Marital status								
Single mother	9.1	1.0 (0.6–1.6)	6.1	2.3 (0.6–8.9)	21.9	1.0 (0.7–1.3)	16.0	1.5 (0.6–3.7)
With partner	8.9	*p = *.951	2.7	*p = *.234	22.7	*p = *.783	10.9	*p = *.414
Three or more siblings								
Yes	11.7	1.4 (0.9–2.0)	4.8	1.8 (0.7–4.8)	22.5	1.0 (0.8–1.3)	15.9	1.5 (0.8–2.7)
No	8.5	*p = *.106	2.7	*p = *.255	22.7	*p = *.951	10.7	*p = *.199
Family income								
Lowest quintile	11.4	1.4 (1.0–1.9)	5.2	2.1 (1.2–3.7)	26.3	1.2 (1.0–1.5)	13.9	1.4 (0.9–2.0)
Second-fifth quintile	8.3	*p = *.066	2.5	*p = *.013	21.6	*p = *.069	10.3	*p = *.123
**Number of sociodemographic risks**		**1.2 (1.1–1.4)** ***p *=* *.007**		**1.7 (1.2–2.4)** ***p *=* *.001**		**1.1 (1.0–1.2)** ***p = *.186**		**1.3 (1.0–1.6)** ***p *=* *.017**

Note: % = row per cent of children reporting violence; RR = risk ratio; CI = confidence interval; N/A = Not applicable given zero cell count for violent females in ALSPAC with maternal age < 20.

Numerous perinatal and sociodemographic characteristics predicted increased risk for conduct problems within each study, for both females and males (Table2). Also, the cumulative number of perinatal and sociodemographic risk factors predicted conduct problems in both studies and for both sexes. For females, there was no evidence that these associations differed between Pelotas and ALSPAC (all interaction tests *p *>* *.10). However, for males, associations were significantly weaker in Pelotas than in ALSPAC for the following variables: maternal smoking in pregnancy (interaction *p *=* *.004), maternal age (interaction *p *=* *.059), single mother (interaction *p *=* *.058), cumulative number of perinatal risk factors (interaction *p *=* *.052) and cumulative number of sociodemographic risk factors (interaction *p *=* *.019).

Several risk factors were associated with violence (Table3): unplanned pregnancy (Pelotas females, ALSPAC females), mother smoked in pregnancy (Pelotas females, ALSPAC males), alcohol use in pregnancy (Pelotas males), low maternal education (Pelotas females, ALSPAC females) and low family income (ALSPAC females). Among females in both studies and males in ALSPAC, the cumulative number of perinatal and sociodemographic risk factors was also associated with violence; however, this was not true among Pelotas males. For females, associations between risk factors and violence were weaker in Pelotas than in ALSPAC for alcohol use in pregnancy (interaction *p *=* *.082) and the number of sociodemographic risk factors (interaction *p *=* *.046). For males, associations were weaker in Pelotas than in ALSPAC for maternal smoking in pregnancy (interaction *p *=* *.040), and the number of sociodemographic risk factors (interaction *p *=* *.082).

Because the literature suggests that biosocial interactions may predispose to violence (Raine, 2002, 2013), we tested whether an interaction between the number of perinatal risk factors (a proxy for biological vulnerability) and the number of sociodemographic risk factors (a proxy for social adversity) predicted violence. No interaction was significant in either study for either sex.

### Do perinatal and sociodemographic risk factors explain the increased risk for conduct problems and violence in Pelotas compared with ALSPAC?

Table4 shows the extent to which cumulative exposure to risk factors explained higher rates of conduct problems and violence in Pelotas compared with ALSPAC. In Model 1, the ratio of conduct problems between Pelotas and ALSPAC was 3.88 for females and 3.55 for males, adjusting only for age at outcome assessment. Models 2, 3 and 4 show reductions in Pelotas–ALSPAC ratios when accounting for cumulative exposure to perinatal and sociodemographic risk factors. The reduction in the size of ratios from Model 1 to Model 4 was 19% for females and 17% for males, indicating that almost a fifth of the cross-national difference in rates of conduct problems was explained by cumulative exposure to risk factors. Considering violence, the reduction in the Pelotas–ALSPAC ratio between Model 1 and Model 4 was 15% for females and 8% for males. Note that, for violence, the confidence intervals in Model 4 overlap with the risk ratio in Model 1, so the magnitude of these changes must be considered tentatively.^3^

**Table 4:** Cross-national differences in conduct problems and violence: Ratio between Pelotas and ALSPAC estimated in Poisson regression models

	MODEL 1	MODEL 2	MODEL 3	MODEL 4
	No risk factors	Number of perinatal risks factors	Number of sociodemographic risk factors	Number of perinatal & Number of sociodemographic risk factors
Conduct problems Pelotas–ALSPAC ratio (95% CI)				
Females	3.88 (3.28–4.58)	3.27 (2.75–3.90)	3.48 (2.94–4.12)	3.14 (2.64–3.73)
Reduction in ratio	Reference	16%	10%	19%
Males	3.55 (3.11–4.06)	3.11 (2.72–3.57)	3.17 (2.77–3.64)	2.94 (2.56–3.37)
Reduction in ratio	Reference	12%	11%	17%
Violence Pelotas–ALSPAC ratio (95% CI)				
Females	2.41 (1.71–3.39)	2.11 (1.49–3.00)	2.24 (1.59–3.16)	2.05 (1.45–2.91)
Reduction in ratio	Reference	12%	7%	15%
Males	1.83 (1.45–2.31)	1.72 (1.36–2.18)	1.76 (1.38–2.23)	1.69 (1.33–2.14)
Reduction in ratio	Reference	6%	4%	8%

Note: Based on 50 data sets created using multiple imputation of missing data (N = 9,821 females; N = 10,189 males). All models include age at outcome measurement as covariate. CI = confidence interval for ratio; all *p* values for ratios <.001.

## Discussion

This study examined associations between perinatal and sociodemographic risk factors at birth and conduct problems and violence in two large, population-based, prospective studies in Brazil and Britain. Across a range of indicators, Brazilian children were exposed to many more risk factors than British children, emphasising the different starting points for children born in middle- versus high-income settings, with possible repercussions for development (Walker et al., 2007). Given the health burdens associated with conduct problems and violence in many low- and middle-income countries, it is important to test whether risk factor associations identified elsewhere replicate in these settings. We found that many of the same risk factors were significant in both Brazil and Britain. For example, conduct problems were predicted by the following in both contexts: maternal smoking in pregnancy, low maternal education, single mother, low family income, and cumulative exposure to perinatal and sociodemographic risk factors. However, several risk factor associations were significantly weaker in Brazil than in Britain, and prediction of male youth violence in Brazil was particularly poor.

One reason why perinatal factors may be less important for violence in Brazil than elsewhere is the ‘social push’ hypothesis (Raine, 2013), whereby early biological influences are less important in contexts where social influences play a strong role. Although perinatal factors may influence childhood conduct problems in Brazil, youth violence could be primarily influenced by social processes in adolescence, such as gangs, drug and arms trades, disordered schools, weak state infrastructure in poor communities, a culture of violence, and ineffective police and justice systems (Murray, Anselmi, Gallo, Fleitlich-Bilyk, & Bordin, 2013; Murray, Cerqueira, & Kahn, 2013). The only other study of early risk factors for violence in Brazil (based on an older cohort in the same city of Pelotas; Caicedo, Gonçalves, González, & Victora, 2010) also reported few significant risk factors; only family income and maternal skin colour predicted conviction for violence among males to age 25. The lack of association between family income and violence in our current study may reflect the different age at outcome measurement, our use of self-reports (official records could indicate police bias, or more serious criminal behaviour) or reductions in absolute poverty levels between the previous study and the current one.

A novel feature of the current study was examining whether cross-national differences in rates of conduct problems and violence might be explained by risk factors at birth. Greater exposure to perinatal and sociodemographic risk factors in Brazil compared with Britain explained almost one fifth (19% females, 17% males) of the cross-national difference in rates of maternal-reported conduct problems. This is consistent with theory and research on the importance of health and social factors in the first years of life for early onset antisocial behaviour (Liu, 2011; Moffitt, 1993; Raine, 2013). We also found that higher exposure to risk factors in Brazil than in Britain accounted for a nontrivial amount (15%, females, 8% males) of the cross-national difference in rates of violence. It is perhaps remarkable that *any* of the cross-national difference in youth violence might be attributed to risk factors measured at birth, given the significance of macrolevel factors, such as national levels of income inequality and welfare support, highlighted in prior research on cross-national variations in violence (Nivette, 2011). Also, it was surprising that perinatal risk factors explained slightly more of the cross-national differences in rates of conduct problems and violence than sociodemographic factors, and this was true for both females and males. Although it is novel to find that perinatal factors might have anything to do with cross-national differences in violence, nearly all of the difference in rates of violence between Brazil and Britain was *not* explained by risk factors measured at birth, highlighting the critical importance of postnatal influences for understanding and preventing violence.

### Strengths and limitations

A major strength of this study was the collection of prospective data on risk factors, conduct problems and violence, measured with similar instruments at similar ages, in two large, longitudinal, population-based surveys in Britain and Brazil. We are not aware of any prior study that has compared prospectively measured risk factors for violence between such different social settings. Also, although most studies of antisocial behaviour have included only boys, both our Brazilian and British studies included females and males.

The following limitations should be acknowledged. Although in Pelotas, the subsample without crime data was very similar to the majority of participants with crime data, this was not true in ALSPAC. We accounted for missing data as best we could using multiple imputation; also some evidence suggests that that predictive models are quite robust to missing data (Wolke et al., 2009). However, we cannot rule out the possibility that results might have been different if there were no missing data. Our study did not include all possible perinatal risk factors, such as birth complications, and did not include postnatal measures, for example nutrition, parenting practices or quality of the home environment. If additional measures were available it might have been possible to better explain cross-national differences. We must also be cautious about the magnitude of cross-national differences in violence explained by variables in this study, given that confidence intervals were wide in relevant statistical models. Another limitation of the study is that the instruments used to measure risk factors were not identical in Pelotas and ALSPAC. Although we tried to maximise comparability between studies by selecting the most objective indicators we could, it is possible that subtle measurement differences or cultural meanings of the variables might have reduced comparability and limited explanation of cross-national differences in outcomes. Another possible reason why risk factors explained only a limited extent of the cross-national differences, is that elevated rates of conduct problems and violence observed in Brazil might primarily reflect reporting bias. We find this very unlikely with respect to youth violence, given that official records in both countries corroborate the higher rates of violence reported in Brazil (Murray, Cerqueira et al., 2013). However, there is unresolved debate about whether Brazilian children actually have such high levels of conduct problems, or whether Brazilian parents tend to overreport problems in short questionnaires (Murray, Anselmi et al. 2013). To help assess this possibility, future studies should include additional measures of child behaviour problems (e.g. also diagnostic assessments). Finally, while both studies used large community populations, neither used national samples and results reflect each local population.

## Conclusions

We conclude that perinatal and sociodemographic risk factors measured at birth are more prevalent in Brazil than in Britain, and have some predictive power within each context. However, almost none predict male youth violence in Brazil, and such early life influences explain only a modest amount of the higher rates of conduct problems and violence in Brazil compared with Britain.

## Key points

- Conduct problems and violence cause a large health burden, especially in low- and middle-income countries in Latin America and Africa; however, almost all major research projects have been conducted in high-income countries.
- This is the first study comparing risk factors for conduct problems and violence between a middle-income and high-income country.
- Brazilian children were exposed to many more risk factors at birth than British children.
- Perinatal and sociodemographic risk factors predicted child conduct problems and violence in both contexts, but not male violence in Brazil.
- Exposure to early life risk factors explained a modest amount of the cross-national difference in rates of conduct problems and violence between Brazil and Britain.

## Notes

1 Our calculations using United Nations population data and Global Burden of Disease 2010 Study homicide data.

2 By definition, low family income (lowest quintile within each study) had similar rates in Pelotas and ALSPAC.

3 We also ran similar models using individual risk factors, but these did not explain Pelotas–ALSPAC ratios as well as cumulative numbers of risk factors.

## Supporting Information

**Table S1.**Perinatal and sociodemographic characteristics at birth. **Table S2.**Cross-national differences in conduct problems and violence.
