# Supplementary material for: Perinatal and sociodemographic factors at birth predicting conduct problems and violence to age 18 years: comparison of Brazilian and British birth cohorts
Source: J Child Psychol Psychiatry. 2014 Dec 4;56(8):914–22. doi: 10.1111/jcpp.12369 (PMC4508966; doi:10.1111/jcpp.12369)
Supplement: Supplementary file 1 — Table S1.Perinatal and sociodemographic characteristics at birth. Table S2.Cross-national differences in conduct problems and violence. [file jcpp0056-0914-sd1.docx]

# Supporting information for Perinatal and socio-demographic factors at birth predicting conduct problems and violence to age 18 years: comparison of Brazilian and British birth cohorts, Murray et al.

# Table S1. Perinatal and socio-demographic characteristics at birth by availability of crime data at age 18

|  | PELOTAS Total Sample | | ALSPAC Total Sample | |
| --- | --- | --- | --- | --- |
|  | Valid Crime Data | No Crime Data | Valid Crime Data | No Crime Data |
|  | N = 3,618 | N = 1,631 | N = 4,012 | N = 10,750 |
|  | % | % | % | % |
| **Unplanned pregnancy** | *p*=.447 | | *p*<.001 | |
| Yes | 55.9 | 57.1 | 24.61 | 33.2 |
| No | 44.1 | 42.9 | 75.4 | 66.8 |
| **Ever smoked in pregnancy** | *p*=.239 | | *p*<.001 | |
| Yes | 32.9 | 34.5 | 15.9 | 28.0 |
| No | 67.1 | 65.5 | 84.1 | 72.0 |
| **Regular alcohol use in pregnancy** | *p*=.584 | | *p*=.287 | |
| Yes | 5.3 | 5.0 | 8.4 | 7.8 |
| No | 94.7 | 95.0 | 91.6 | 92.2 |
| **Urinary infection in pregnancy** | *p*=.818 | | *p*<.001 | |
| Yes | 33.7 | 33.4 | 11.8 | 14.8 |
| No | 66.3 | 66.7 | 88.2 | 85.2 |
| **Intrauterine growth restriction** | *p*=.366 | | *p*=.002 | |
| Yes | 90.8 | 10.0 | 7.5 | 9.2 |
| No | 9.2 | 90.0 | 92.5 | 90.8 |
| **Preterm birth (< 37weeks)** | *p*=.007 | | *p*=.853 | |
| Yes | 10.6 | 13.2 | 5.0 | 6.8 |
| No | 89.4 | 86.8 | 95.0 | 93.2 |
| **Maternal** **age** | *p*=.731 | | *p*<.001 | |
| <20 | 17.6 | 17.2 | 1.6 | 5.8 |
| ≥20 | 82.4 | 82.8 | 98.4 | 94.2 |
| **Maternal education** | *p*=.003 | | *p*<.001 | |
| Low | 26.8 | 30.8 | 11.0 | 24.0 |
| Medium-high | 73.2 | 69.2 | 89.0 | 76.0 |
| **Marital status** | *p*=.397 | | *p*<.001 | |
| Single mother | 12.1 | 12.9 | 98.4 | 97.2 |
| With partner | 87.9 | 87.1 | 1.6 | 2.8 |
| **Three or more siblings** | *p*=.191 | | *p*<.001 | |
| Yes | 13.5 | 14.8 | 4.1 | 6.6 |
| No | 86.5 | 85.2 | 95.9 | 93.4 |
| **Family income** | *p*=.006 | | *p*<.001 | |
| Lowest quintile | 19.1 | 22.4 | 14.4 | 23.0 |
| Second-fifth quintile | 81.0 | 77.7 | 85.7 | 77.0 |

Note. Column percents. *p* values refer to Chi-square tests comparing percentages within Pelotas groups and within ALSPAC groups

Table S2. Cross-national differences in conduct problems and violence: Ratio between Pelotas and ALSPAC estimated in Poisson regression models using complete cases

|  | **MODEL 1** | **MODEL 2** | **MODEL 3** | **MODEL 4** |
| --- | --- | --- | --- | --- |
|  | **No risk factors** | **Number perinatal  risks factors** | **Number socio-demographic  risk factors** | **Number perinatal &**  **Number socio-demographic**  **risk factors** |
| **CONDUCT PROBLEMS**  **PELOTAS-ALSPAC Ratio** |  |  |  |  |
| Females (95% CI) | 4.81 (3.96-5.84) | 3.90 (3.19-4.76) | 4.08 (3.34-4.98) | 3.54 (2.89-4.33) |
| Reduction in ratio | Reference | 19% | 15% | 26% |
|  |  |  |  |  |
| Males (95% CI) | 4.29 (3.64-5.06) | 3.73 (3.15-4.43) | 3.68 (3.11-4.37) | 3.36 (2.83-4.00) |
| Reduction in ratio | Reference | 13% | 14% | 22% |
|  |  |  |  |  |
| **VIOLENCE**  **PELOTAS-ALSPAC Ratio** |  |  |  |  |
| Females (95% CI) | 3.88 (2.18-6.93) | 3.21 (1.77-5.81) | 3.20 (1.78-5.77) | 2.82 (1.55-5.15) |
| Reduction in ratio | Reference | 17% | 18% | 27% |
|  |  |  |  |  |
| Males (95% CI) | 1.95 (1.46-2.61) | 1.85 (1.37-2.48) | 1.84 (1.37-2.4) | 1.78 (1.31-2.40) |
| Reduction in ratio | Reference | 5% | 5% | 9% |

Notes. For conduct problems, N = 4,611 females and N = 4,530 males. For violence, N = 3,009 females and 2,644 males. All models include age at outcome measurement as covariate. CI = confidence interval for ratio; all *p* values for ratios < .001
